# The Influence of Sandblasting Process Parameters of Aerospace Aluminium Alloy Sheets on Adhesive Joints Strength

**DOI:** 10.3390/ma14216626

**Published:** 2021-11-03

**Authors:** Izabela Miturska-Barańska, Anna Rudawska, Elżbieta Doluk

**Affiliations:** Department of Production Engineering, Faculty of Mechanical Engineering, Lublin University of Technology, Nadbystrzycka 36, 20-618 Lublin, Poland; a.rudawska@pollub.pl (A.R.); e.doluk@pollub.pl (E.D.)

**Keywords:** shear strength, single-lap adhesive joints, EN AW 2024 T3 aluminium alloy, sandblasting

## Abstract

In this study, the influence of sandblasting process parameters as a surface preparation method on the strength of single-lap adhesive joints of EN AW 2024 T3 aerospace aluminium alloy sheets was determined. Eleven sets of sandblasting parameters were used, which were determined according to a determined experimental plan. The variable factors in the sandblasting process were pressure, nozzle distance, and workpiece displacement speed. The sand jet incidence angle was constant. Garnet 80 E+ was the abrasive material that was used. The joints were made using an epoxy adhesive composition of Epidian 5 epoxy resin and a PAC curing agent. The influence of the surface preparation method on the surface roughness and contact angle to determine the surface free energy was evaluated. The shear strength of the adhesive joints was also determined, which finally allowed the evaluation of the applied surface treatment variants. The obtained results were subjected to statistical analysis, which indicated that the highest shear strength of the adhesive joints was obtained for samples whose surfaces were treated by sandblasting at parameter configurations in which the pressure was 5–6 × 10^5^ Pa; the distance between the nozzle and the sandblasted surface should not be greater than 97 mm, and the speed at which the workpiece moves in relation to the nozzle should not be greater than 75 mm/min.

## 1. Introduction

One of the most developed methods of joining materials that has been in recent times is adhesive bonding, the key advantage of which is the possibility of joining elements made of materials that are characterized by various mechanical, physical, and chemical properties [1,2,3,4]. Through adhesive bonding, it is possible to make structures that are much larger than they can be made as a single element or that can be transported as a single unit [5]. The bonding technology also contributes to the design and the manufacture of lightweight yet strong structures with specific properties [6]. Because of this versatility, this technology is used in a wide range of industries, such as the aerospace, automotive and rail, and civil engineering industries as well as other industrial sectors where an effective high strength-to-weight ratio is important for the development of the innovative structures [7,8,9,10,11,12,13,14,15]. As Messler describes in his publications [16,17], adhesive bonding technology creates the possibility of replacing other bonding methods without causing microstructural changes in the joined elements. A number of the advantages resulting from the use of this joining method are due to the fact that the adhesive joints can also be successfully applied in the electronics and construction industries as well as in medicine and material engineering [9,18,19]. As indicated by the authors of Saboori et al. [20] and Ziółkowski and Dyl [21], structural bonding may be a worthwhile alternative in repair and sealing processes, where regenerating parts in the repairs of machine structure elements, allows costs being able to be reduced. During vehicle construction, adhesive technology can be used to fix linings, brake pads, glass panes, joining elements of door skins, engine covers, and the boot [13,14,22,23]. Adhesive technology is also used in bus manufacturing due to the excellent damping properties of the adhesives. An important share is also recorded in the construction of self-supporting vehicle bodies [13,24]. These adhesives are also used to seal internal combustion engines, differentials, and transmissions and can also be used to reinforce thrust-bearing contact surfaces. Structural adhesive bonding is also now widely used in the construction of aircraft airframes [25,26,27].

The correct execution of the bonding process consists of several steps [12,15,28]. The correct implementation of the individual stages of the technological process affects the correct execution of adhesive joints that are characterized by a specific strength. The most important and initial stage is the preparation of the surface of the joined materials. Surface preparation has a very large influence on the strength and resistance of the adhesive joints. When choosing the appropriate surface treatment method, it is necessary to know the type, structure, and properties of the adherends [15,29,30,31].

In this study, aerospace aluminium alloys were used. Aluminium and aluminium alloy surfaces are usually processed chemically and electrochemically. Anodizing, chromating, and phosphating are frequently used [3,10,11,22,31,32,33,34]. However, due to the availability of materials and the safety of the process, the most commonly used process is mechanical treatment [35,36,37]. Mechanical treatment is one of the most frequently used methods for the surface treatment of construction materials to be used in the bonding process [32,38,39,40,41]. One of the reasons for the use of mechanical treatment is to clean various impurities from the surface that have managed to become stuck to the surface through various means (e.g., oxides, fine impurities) as well as to change the geometric structure of the adherend surfaces. These are important activities that are related to obtaining the appropriate (favorable) stereometric structure of the surface and that influence the adhesive properties of the adherend surfaces. As a result, the surface is geometrically developed, and this affects the increase in mechanical adhesion, which is related to the increase in the active area where the adhesive is in contact with the adherent [42]. When using mechanical processing, the correct choice of abrasive grain size is crucial. Too small a grit size can cause the contaminants to be smeared across the surface, whereas coarse grit sizes create deep scratches and craters on the surface, which can cause changes in the properties of the surface layer [43,44]. The machining process should be followed by a final degreasing process to remove dust, dirt, grease, and other contaminants. Degreasing can be accomplished by using trichloroethylene, acetone, carbon tetrachloride, extraction gasoline, or other agents to emulsify the contaminants.

When considering machining methods, various methods that have been the subject of analysis in scientific works can be distinguished, including abrasive blasting (including sandblasting), shot blasting, shot blasting, grinding, and others [45,46,47,48,49]. Among the mechanical treatments, sandblasting is recommended and is the most favorable, while the coarse-grained abrasive cloth method is the least favorable. During sandblasting, it is to the use of an aluminium oxide, silicon carbide, and a quartz as the abrasives which is recommended due to their irregular shape and the sharp edges, making them effective in imparting a certain roughness to the treated surface [47,50,51]. Abrasives in the form of glass beads, porcelain beads, or metal shot should not be used on aluminium and its alloys, as they only cause surface crushing [52].

The aim of this study was to determine the influence of the parameters of the sandblasting process as a surface preparation method on the strength of the single-lap adhesive joints of EN AW 2024 T3 aerospace aluminium alloy sheets. The adhesive joints were made using an epoxy adhesive composition. The tests also included surface roughness and contact angle measurements to determine the surface free energy. The shear strength of the adhesive joints was also determined. The obtained test results were subjected to statistical analysis.

## 2. Materials and Methods

### 2.1. Adherend

In this study, an EN AW 2024 T3 aluminium alloy was used. This material is characterized by lower corrosion resistance and poorer weldability than other aluminium alloys, but it contains a high amount of copper and has very high strength—compared to AW2014, for example—and high fatigue strength. The chemical composition of the alloy that was used is given in Table 1. This material is most often used in structures requiring high strength but is also used where the risk of corrosion is low. It is used in parts where a high strength-to-weight ratio is required; therefore, it is used in the construction of aircraft equipment, gears and shafts, screws, computer parts, clutches, hydraulic valve parts, rocket and munitions parts, pistons, worm gears, and orthopedic equipment. Table 2 presents the mechanical properties of the material used in this study.

The specimens used in the study were cut from a sheet of a plate with a thickness of 2.00 ± 0.12 mm to dimensions of 101.60 ± 0.25 mm × 177.80 ± 3.17 mm by means of a hydroabrasive jet using a Waterjet Eckert Combo portal cutting machine (Eckert AS Ltd., Legnica, Poland). The cutting process speed was 200 mm/min at a water pressure of $3500\times 10^{5}$ Pa. The distance between the nozzle and the material being cut was 3 mm, and the abrasive flow rate during the cutting process was approximately 0.4 kg/min. The abrasive used was Garnet 80 E+ sand. Subsequently, two holes with a diameter of 2.5 mm were drilled into each of the cut sheets to grind the fixing pins, which made it possible to assemble the adhesive panels in a defined geometry while maintaining a constant overlap length.

### 2.2. Surface Preparation Methods

**Figure 1:**
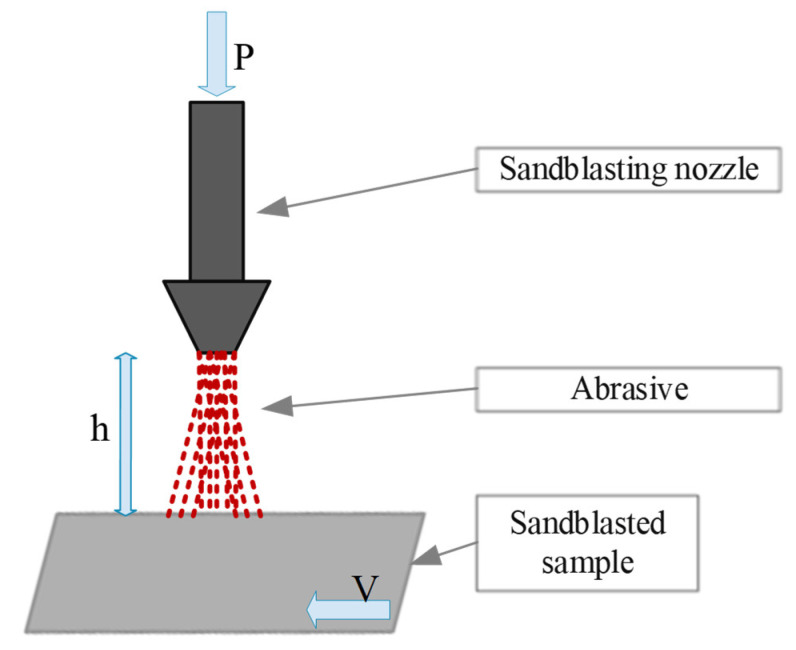
Sandblasting process diagram.

The surface of the cut sheets was prepared for the bonding process by sandblasting and degreasing the surface with acetone. The process of sandblasting the samples for adhesive bonding was carried out on a specially modernised cabin sandblaster (Cormak, Siedlce, Poland). The sandblasted sample was placed in a holder and was driven by an electric motor so that a constant speed of nozzle movement over the sample was achieved. The pressure was set on a regulator, and parameters such as the angle of incidence of the sand jet and the distance of the nozzle from the workpiece were achieved by keeping the nozzle and the sample stationary. Garnet 80 E+ sand was used in the sandblasting process. A diagram of the sandblasting process is shown in Figure 1. The sample was moved at a constant speed V and was sandblasted with the nozzle placed at a distance h from the sandblasted surface using a specific pressure P.

In order to select the optimal sandblasting parameters, tests were carried out according to the Hartley PS/DS-P:Ha_3_ determined selection plan [55]. The Hartley plan allowed for a significant reduction in the number of experiments that needed to be carried out. The basic principle of creating poliselection plans is the deliberate selection of the combination of input values (within the previously assumed range) in such a way that it is possible to obtain the required scientific information with limited effort, i.e., a relatively small number of measurements. This plan belongs to the static, determined, policy-selective plans for three input quantities, where five different values are used for each input quantity. The test plan included different combinations of sandblasting parameters in which the variables were sample displacement speed, sandblasting pressure, and nozzle distance from the sample surface. The angle between the sample and the jet direction was assumed to be constant at 90°. The number of sample displacements was also assumed to be two. To perform the calculations to determine the parameter sets according to the Hartley Plan, it was necessary to establish the minimum and maximum values achievable for each parameter, which are summarized in Table 3. The minimum and maximum values of the parameters were determined on the basis of our own preliminary tests to check the capabilities of the machine on which the sandblasting process was carried out.

The parameter sets determined according to the test plan are presented in Table 4.

The samples were sandblasted according to the specified parameters and were then cleaned of dust and any contaminants in an acetone bath. The samples were immersed in the acetone bath for 20 min at 23 ± 2 °C, after which the surfaces were wiped with a cleaning rag, cleaned a second time, and allowed to dry internally for 10 min.

### 2.3. Shape and Dimension of Adhesive Joints and Specimen Preparation Conditions

Single-layer adhesive joints of aluminium alloy sheets were prepared using an epoxy adhesive composition of Epidian 5 epoxy resin (CIECH S.A., Sarzyna, Poland) and the PAC curing agent (CIECH S.A., Sarzyna, Poland), which were mixed in a stoichiometric ratio of 100:80 (identification of composition—E5/PAC/100:80). Epidian 5 is a pure form of epoxy resin, which is a product of the reaction of bisphenol A with epichlorohydrin. It is characterised by excellent adhesion to most plastics, chemical resistance as well as resistance to aggressive environmental factors, and good electrical properties [56,57]. Epidian 5 resin and compositions based on it are used in the manufacture of glass fiber laminates, joining metals, ceramics, and thermosetting plastics. Adhesives prepared on the basis of this resin are also used in building structures that are used as anti-corrosive and electro-insulating coatings. The performance properties of the resin used in this study are presented in Table 5.

PAC curing agent (CIECH S.A., Sarzyna, Poland) (fatty acids, C18-unsaturated, dimers, polymeric reaction products with triethylenetetramine) is used to harden liquid epoxy resins. This hardener causes the flexibility and impact strength of the composition to increase, which is why it is used for joints that are exposed to deformations, e.g., in boatbuilding to join wooden elements or elements made of polyester-glass laminates; to join rubber with metal, thin sheets and plywood; and to pour elements in electrical engineering and electronics. The PAC curing agent belongs to a group of slow curing agents. Full cure is achieved in 7–14 days. The functional properties of the hardener used in this study are shown in Table 6.

Adhesive composition was prepared straight before the bonding process. The components of the mixtures were carefully weighed using a KERN CKE 3600-2 laboratory scale (Kern, Albstadt, Germany) with a measurement accuracy of 0.01 g. Before the mixing process, the epoxy resin was preheated to 50 °C to reduce its viscosity. The epoxy resin heating stage was conducted using an electric heater—DEPILUX 400 (Activ, Wroclaw, Poland)—with the power of 100 W, which allows for the smooth regulation of the liquid heating from 45 to 105 °C. The temperature of the heated epoxy resin was monitored using an electronic thermometer (Amarell Electronic, Kreuzwertheim, Germany) with the measuring range of −50 to 200 °C and with the measuring accuracy of ±0.1 °C. Then, the heated epoxy resin was mixed with a mechanical mixer Güde GTB 16/5 A (Güde, Wolpertshausen, Germany) equipped with a propeller mixer. The mixing process used a speed of 460 rpm lasted for 2 min. Next, the adhesive compositions were deaerated for 2 min in order to remove any gas bubbles that had formed as a result of mixing the components. The finished adhesive compositions were applied to the surfaces to be bonded using a roller for adhesive application, which made it possible to achieve a homogeneous joint thickness across the entire adhesive surface. In the next stage, the elements were joined together. The adopted curing conditions for the adhesive joints are presented in Table 7.

**Figure 2:**
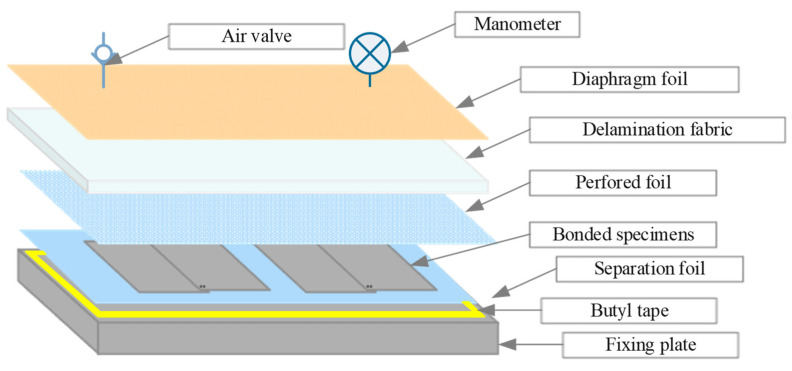
Layout of vacuum bag components with bonded specimens.

**Figure 3:**
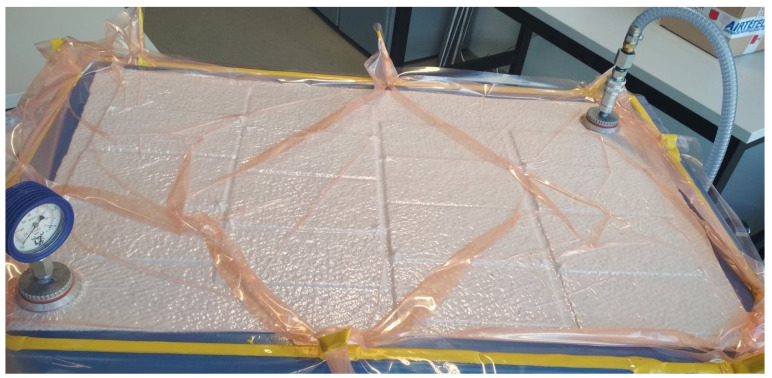
View of bonded specimens in a vacuum bag.

Constant pressure throughout the curing period for the adhesive joints was ensured by the vacuum bag method, which was implemented using a SVAGG vacuum pump (Schunk, Lauffen/Neckar, Germany). The vacuum bag method is illustrated in Figure 2. Pictures of the real joint-making process are shown in Figure 3.

**Figure 4:**
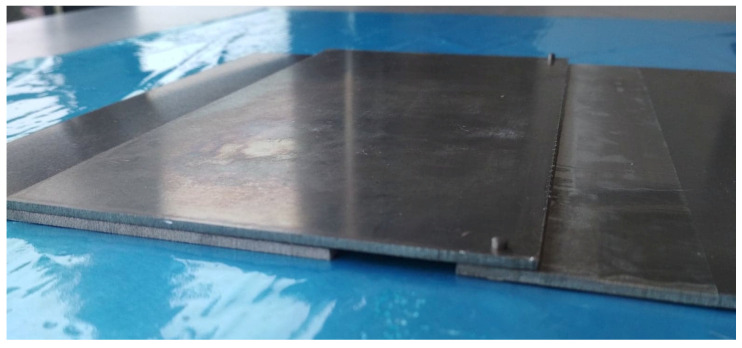
Method of assembling the bonded panels used in the tests.

The adhesive joints used in the tests were prepared in accordance with the requirements of the ASTM D1002 standard [59] in the form of panels. The general appearance of the prepared panels with a fixed overlap length, which, according to the standard, is 12.7 mm, is shown in Figure 4.

**Figure 5:**
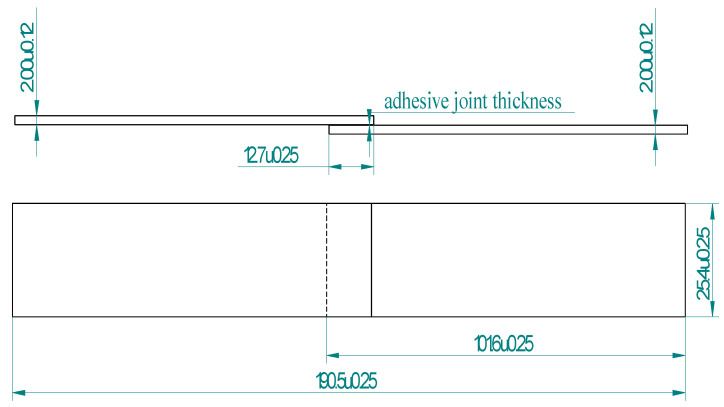
Single-lap adhesive bonding used in the tests performed in accordance with the ASTM D1002 standard.

After the joint seasoning time, the panels were cut on a Waterjet Eckert Combo machine using the same parameters as those used for sheet cutting. From one panel, five samples were obtained for each sandblasting variant. The geometry and dimensions of a single adhesive specimen intended for testing are shown in Figure 5.

**Figure 6:**
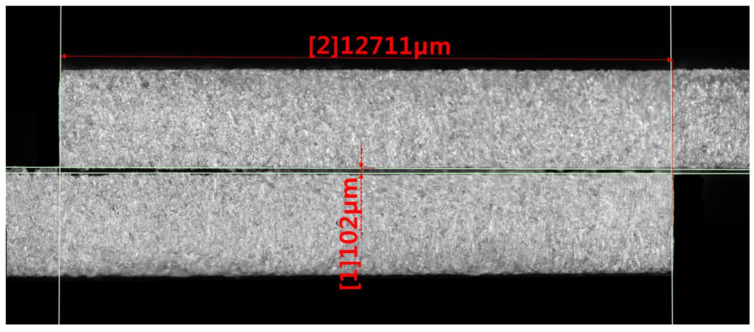
Sample images of overlap joint taken with the Keyence VHX-5000 microscope.

The overlap length and adhesive bond thickness were measured using a Keyence VHX-5000 digital microscope (Keyence, Itasca, IL, USA). Figure 6 shows an example photo of the measurement of the overlap length and adhesive joint thickness of the tested adhesive joints.

The average adhesive joint thickness was 0.100 ± 0.025 mm.

### 2.4. Surface Roughness

Before the bonding process, the surface roughness and 3D topography features of the EN AW 2024 T3 aluminium alloy samples were evaluated after the sandblasting process was complete using a T8000 RC120-400 contour, roughness, and 3D topography measuring device (Hommel-Etamic, Berlin, Germany). Tests were conducted in accordance with the EN ISO 25178 standard [60]. A TKU300 measuring tip was used in the study. The measuring range was 80 μm. The tests were conducted at a speed of 0.50 mm/s. The sampling length was set to lr = 0.8 mm. The area that was scanned included a 4.8 mm × 4.8 mm section of the surface, and the roughness profile parameters were determined from 241 measurements. The following amplitude parameters of the roughness profile and surface topography were analysed: Ra—arithmetic mean deviation of roughness, Rz—highest profile height, Sa—arithmetic mean surface height, and Sz—maximum surface height. Ten measurements were taken for each sample.

### 2.5. Surface Energy

Before the bonding process, the contact angle of the aluminium alloy sheet surface was also measured after the sandblasting process to determine the surface free energy. The surface free energy was determined using the Owens–Wendt method, which is based on direct measurements of the contact angle [42,61,62]. The Owens–Wendt method is based on the determination of two components: polar and dispersion surface free energy, according to Equation (1) [47,63]:

(1) $$\gamma_{s}=\gamma_{s}^{d}+\gamma_{s}^{p}$$

where $\gamma_{s}$—surface free energy (SFE), $\gamma_{s}^{d}$—dispersion component of SFE, $\gamma_{s}^{p}$—polar component of SFE.

To determine the total surface free energy, two measuring liquids (non-polar and polar) with known polar and dispersion component values were used to determine the surface free energy. Distilled water and diiodomethane CH_2_I_2_ were used as measuring liquids, and the droplet size that was used was approximately 2 μL. The polar component of the distilled water was 51 mJ/m^2^ and had a total surface free energy equaling 72.8 mJ/m^2^ [42,62]. The individual components of the surface free energy of diiodomethane equaled dispersion—48.6 mJ/m^2^, and polar—2.4 mJ/m^2^ [63], respectively. The components $\gamma_{s}^{d}$ and $\gamma_{s}^{p}$ for the surface free energy of the adherend surface could be calculated from the equations presented in [42].

The droplet size of the measuring liquids was approximately 2 μL. There were 15 measurement respetitions on the measurement on each aluminium alloy sample with each measuring liquid. The reading of the contact angle value was made 5 s after the liquid drop was formed. Contact angle measurements were made at a temperature of 21 ± 1 °C and at an air humidity of 30 ± 1%. The tests were conducted using a PGX pocket goniometer (Kontech, Lodz, Poland).

### 2.6. Strength Test

Strength tests of single-lap adhesive joints of EN AW 2024 T3 aluminium alloy sheets under shear stress were conducted on a Zwick Roell Z150 testing machine (Zwick/Roell, Ulm, German) according to the ASTM D1002 standard [49]. This is a standard for testing the apparent shear strength of single-lap specimens by tensile loading. The crosshead displacement during the test was 1.5 mm/min at an initial force of 5 N.

## 3. Results and Discussion

### 3.1. Surface Roughness

**Figure 7:**
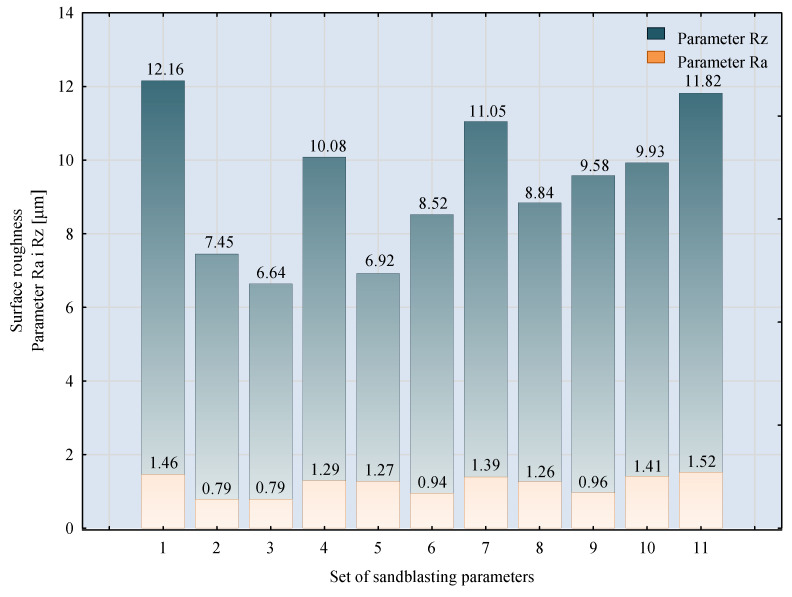
Ra and Rz surface roughness parameters of EN AW 2024 T3 aluminium alloy samples after sandblasting.

The results of the surface quality assessment for the samples after the sandblasting process are presented below (Figure 7 and Table 8).

Figure 7 presents the results of surface roughness testing of the samples after sandblasting. The highest average values of the Rz and Ra parameters were obtained for 1, 7, and 11 sets of the sandblasting parameters (described in Table 4). In the mentioned variants of the process, the sandblasting pressure was 5 and 6 bar, the distance of the nozzle for one set of parameters was 69 mm, and for the other two, it was 97 mm, while the sandblasting speed for one sandblasting variant was 62 mm/min, for seven set of parameters it was 53 mm/min, and for ten variants, it was 75 mm/min. For comparison, the surface topography of the samples after sandblasting is presented in Table 8.

From the surface topographies shown in Table 8, it can be seen that the sandblasting process did not create any deformation on the surfaces of the samples that were to be bonded. This could only be observed on the uniformly distributed surface depressions, which are desirable in the adhesive process since in the next stage, the adhesive penetrates into the created depressions, anchoring itself and forming mechanical bonds between the surfaces. Analyzing the surface topography parameters Sa and Sz, it can be observed that the distribution of the obtained results is similar to that of the surface roughness profile parameters Ra and Rz.

### 3.2. Surface Energy

**Figure 8:**
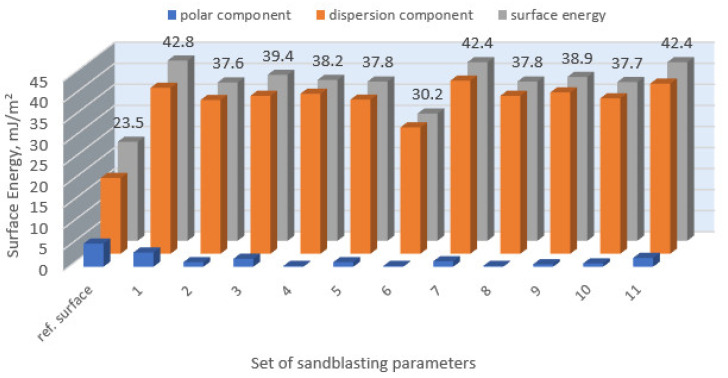
Surface free energy of EN AW 2024 T3 aluminium alloy sheet samples after sandblasting.

Figure 8 presents the calculated surface free energy results for the sandblasted surfaces.

For comparison, the surface free energy results for a reference aluminium alloy surface not treated with sandblasting are also included. As it can be seen, the untreated surface is characterised by a significantly lower surface free energy value (23.5 mJ/m^2^). Such a surface is characterised by lower wettability, which may consequently lead to the weakening of adhesive bonds in the adhesive joint. As it can be seen, the highest surface free energy value can be observed in the sample surfaces that were subjected to the 5th sandblasting method (42.8 mJ/m^2^) as well as to the 7th and 11th set of sandblasting parameters (in both cases 42.4 mJ/m^2^). The results obtained were statistically processed. The statistical analysis of these three values showed no significant differences at the adopted significance level α = 0.05. The lowest surface free energy value was observed in the case of the 6th sandblasting method (30.2 mJ/m^2^). As an example, Table 9 shows the appearance of the droplets for the sandblasting methods described as well as the values of the contact angles for all of the surfaces that were analysed.

### 3.3. Strength Test Results

**Figure 9:**
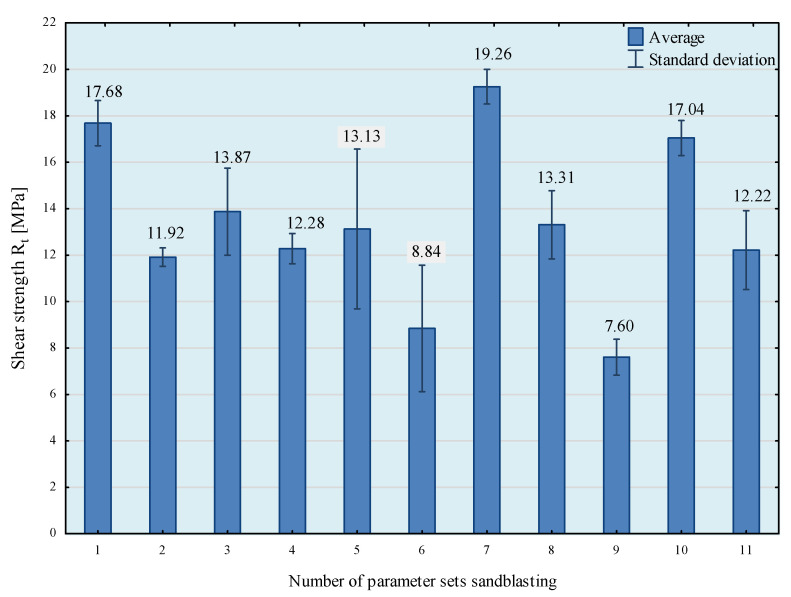
Results of shear strength tests of EN AW 2024 T3 aluminium alloy sheet adhesive joints as a function of sandblasting parameters.

The obtained results of the shear strength tests of adhesive joints depending on the parameters of the surface preparation treatment of the specimens are shown in Figure 9.

From the results obtained here, it can be seen that the highest shear strength (mean value) was obtained for the samples whose surfaces were prepared according to the 7th set of sandblasting parameters. In order to be able to analyse the test results in more detail, a statistical analysis of the obtained results was conducted. The results of the Shapiro–Wilk test are summarised in Table 10.

Based on the results obtained here, it can be noted that the conditions for a normal distribution were not met in any of the groups (in group 3—*p* < 0.05). Therefore, in a later part of the study, a post hoc test was performed where homogeneous groups were determined. The results of this test are presented in Table 11.

When analyzing the results obtained here, it can be observed that group 1 contains the averaged results of three groups: sandblasting parameter sets 7, 1, and 10. However, in the case of adhesive joints whose surfaces were prepared using sandblasting parameter set 7, the highest result repeatability was also obtained (standard deviation of 3.9%). The lowest shear strengths were obtained in the case of sandblasting methods 6 and 9 for the surfaces of the bonded samples.

## 4. Discussion

Some machining methods are not only used to treat the surfaces of construction materials before adhesive processes but are also used to modify surface properties, such as abrasive blasting. This treatment includes sandblasting, shot blasting, and shot peening. Such a modification may contribute to increasing the fatigue strength of the components that are subjected to high stresses. For example, the machined elements (e.g., after milling, cutting or heat treatment) contain residual tensile stresses. Shot peening transforms these stresses into compressive stress, which significantly extends the service life of these components. The surface subjected to shot peening slightly deforms plastically, which causes a change in the direction and nature of the stresses occurring in the surface layer. This issue was emphasized in many works, e.g., in Al-Obaid [45]. Shot blasting is similar to sandblasting, except that it works using a plasticity mechanism instead of an abrasion mechanism. In turn, the use of surface shot peening [64] and laser shock peening [65] emphasize the importance of plastic deformation in improving the surface properties of various construction materials. Abrasive blasting, which includes sandblasting, can cause plastic deformation that roughens the surface and that can cause significant subsurface grain refinement. In [66], Multigner et al. show that the plastic strain gradient and volume increase associated with α′-martensite-induced deformation is responsible for the development of compressive residual stresses with a maximum value near the surface. The different results are related to the specific morphology of the particles and their specific role in the blasting process. Li, Du et al. present similar conclusions in their work [67].

In order to determine the influence of individual parameters, both roughness and surface free energy, a correlation study analysis was performed. The results of this analysis are summarised in Table 12.

Analyzing the results obtained here, it can be seen that the value of the correlation coefficient oscillates between 0.53 and 0.67 (except for the Rz parameter), which indicates strong linear dependence of the strength on the roughness and surface free energy parameters. The coefficient of determination ranges from 0.30 to 0.44 in all cases, which means that the variation in shear strength is almost 44% and can be explained by the variation in the individual parameters. The significance level p for the t-statistic is only less than 0.05 in the case of the Sz parameter, which means that the correlation coefficient is significantly different from 0. Despite the previously mentioned similar distribution of the roughness profile parameters Ra and Rz with the surface topography parameters Sa and Sz, some discrepancies could be observed in the correlation study. They may have resulted from the fact that the Rz parameter is only determined only in the 2D cross-section in one orientation, while the Sz parameter informs the size of the elevations on the tested surface.

In the conducted studies, it was noticed that the sandblasting parameters have a significant influence on the adhesive joint strength. It was shown in [47] that both the surface roughness and the adhesive properties, such as surface free energy, are more dependent on the type of the abrasive material that is used and not on differences in the sandblasting pressure value. Other factors such as sandblasting agent and its abrasive particle size also influence adhesion. In this work, increasing the ratio of the valley depth to the tip height as a result of changing sandblasting parameters, as evidenced by the change in Ra and Rz surface roughness parameters, has a positive effect on the adhesive joint strength. In turn, Staia et al. [68] emphasized that the sandblasting process allows a favorable adherend surface roughness to be obtained and provides the mechanical anchoring between the adhesive and the adherend surface and also depends on one of the sandblasting parameters, which is the pressure during this treatment. In another work [69], the strength and the surface roughness parameters were compared, and it was noticed that in the case of the adhesive joints in which there is a significant roughness of the surfaces of the adherends, there is a greater relationship between the values: the set of Rz and Rt surface roughness parameters and the adhesive joints strength. Mandolfino et al. [70] indicated that the properties of bonded surfaces obtained after the sandblasting process affect the strength of the adhesive joints and also indicated that the type of the sand and the sandblasting pressure also had an effect on the strength of the adhesive joints.

## 5. Conclusions

An important aspect affecting the strength of the adhesive joints, apart from the proper selection of the adhesive composition, is the proper preparation of the adherend surfaces. In the case of the bonding aluminium alloys, the recommended method is the sandblasting. Studies were carried out on the influence of the sandblasting parameters as a method of the surface treatment on the static strength of EN AW 2024 T3 aluminium alloy adhesive joints. This surface treatment method for the bonding was a variable factor in the tests, while factors such as the type of the adhesive and other bonding conditions remained constant during the tests. The roughness measurements of the treated surfaces and surface free energy measurements were also carried out. Based on the strength results obtained for the adhesive joints of the aluminium alloy sheets, the following conclusions can be drawn:

- Both the properties of the adhesive (in particular its viscosity) and the geometric structure of the adhesion surface, after the application of certain surface treatment methods, significantly influence the formation of the actual adhesive–binder interface, as this ensures relatively the high strength due to, among other things, the significant role of the mechanical adhesion;
- Increasing the ratio of valley depth to tip height as a result of changing sandblasting parameters, as evidenced by the change in Ra and Rz parameters, has a positive effect on the strength of the adhesive joints;
- Sandblasting parameters have a significant influence on the strength of adhesive joints. The selection of appropriate parameters is a key factor for obtaining a well-developed surface. The tests conducted here have determined that the pressure of the sandblasting process should not be less than $5\times 10^{5}$ Pa, the distance of the nozzle from the sandblasted surface should not be greater than 97 mm, and the speed at which the workpiece is displaced in relation to the nozzle should not be greater than 75 mm/min. Exceeding these parameters results in a decrease of the roughness profile parameters as well as the value of the surface free energy and thus in a decrease of the shear strength of the constituted adhesive joints;
- The joints with the highest strength were prepared by abrasive blasting with the following parameters: nozzle distance from the sample—h = 97 mm, blasting speed—V = 53 mm/min, pressure—P = $5\times 10^{5}$ Pa. In the case of these joints the highest result repeatability was also obtained.

## Figures and Tables

**Table 1:** Chemical composition of EN AW 2024 T3 aluminium alloy [53].

The Element	Contents, %
Si	0.1671
Fe	0.2153
Cu	4.0975
Mn	0.4281
Mg	1.4405
Cr	0.0053
Zn	0.0154
Ti	0.0191
Al	93.5699

**Table 2:** Mechanical properties of EN AW 2024 T3 aluminium alloy [53,54].

Mechanical Properties	Value
Tensile strength	447.2 MPa
Yield strength	302.5 MPa
Elongation	16.5%
Hardness	123 HB
Thermal conductivity	170 W/mK
Thermal conductivity	2.78 g/cm^3^

**Table 3:** Range of values for selected parameters of the sandblasting process.

Parameter	Parameter Value
X1—distance of the nozzle from the sample surface	40 ≤ X1 ≤ 155 [mm]
X2—speed of sample displacement	50 ≤ X2 ≤ 100 [mm/min]
X3—sandblasting pressure	$3\times 10^{5}\;$≤ X3 ≤ $7\times 10^{5}$ [Pa]

**Table 4:** Hartley plan PS/DS—P: Ha3 for actual input values.

Number of Parameter Sets Sandblasting	X1	X2	X3
	Distance of the Nozzle from the Sample Surface (mm)	Speed of Sample Displacement (mm/min)	Sandblasting Pressure (Pa)
1	69	62	$6\times 10^{5}$
2	126	62	$4\times 10^{5}$
3	69	87	$4\times 10^{5}$
4	126	87	$6\times 10^{5}$
5	48	75	$5\times 10^{5}$
6	147	75	$5\times 10^{5}$
7	97	53	$5\times 10^{5}$
8	97	96	$5\times 10^{5}$
9	97	75	$3\times 10^{5}$
10	97	75	$6\times 10^{5}$
11	97	75	$5\times 10^{5}$

**Table 5:** Physical and chemical properties of Epidian 5 epoxy resin [56,57].

Properties	Epidian 5 Epoxy Resin
Epoxy number	0.48–0.52 mol/100 g
pH value	approx. 7
Viscosity at 25 °C	20,000–30,000 mPa·s
Density at 20 °C	1.16 g/cm^3^
Flash point	266 °C
Auto-ignition temperature	490 °C
Melting point	30–50 °C
Boiling point initial	not indicated—decomposition

**Table 6:** PAC curing agent functional properties [57,58].

Properties	Polyamide Hardener (PAC Curing Agent)
Viscosity at 25 °C	10,000–25,000 mPa·s
Density at 20 °C	1.10–1.20 g/cm^3^
Amine number	290–360 mg KOH/g
Gel time (for example, for a composition with Epidian 5 at 20 °C, for a 100 g sample)	180 min

**Table 7:** Parameters of curing conditions for adhesive bonds.

Curing Process Parameter	Parameter Value
Pressure	$0.6\times 10^{5}$ Pa
Temperature	23 ± 2 °C
Humidity	23 ± 3%
Time	7 days

**Table 8:** Surface topography of EN AW 2024 T3 samples after sandblasting.

Number of Parameter Sets Sandblasting	Surface Topography	Surface Profile Height Parameters
1	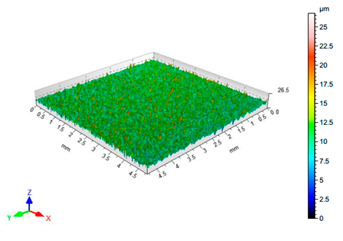	Sa = 1.49 μm Sz = 26.8 μm
2	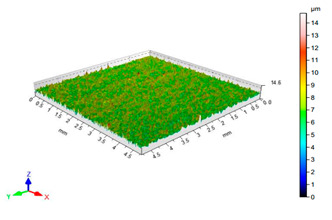	Sa = 0.82 μm Sz = 14.8 μm
3	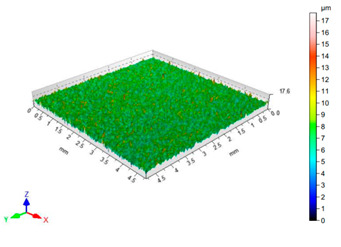	Sa = 0.90 μm Sz = 17.7 μm
4	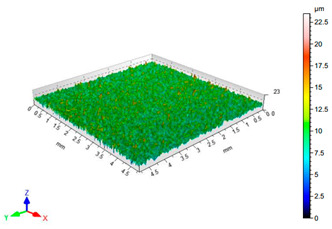	Sa = 1.41 μm Sz = 23.5 μm
5	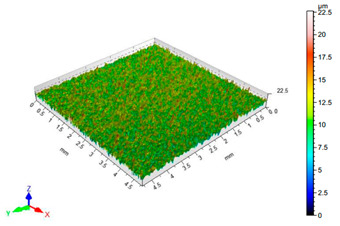	Sa = 1.57 μm Sz = 22.6 μm
6	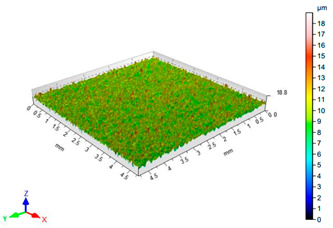	Sa = 0.97 μm Sz = 19.0 μm
7	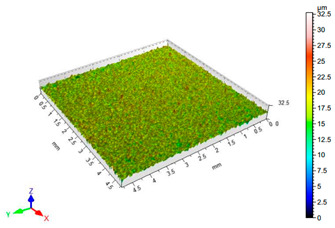	Sa = 1.43 μm Sz = 32.8 μm
8	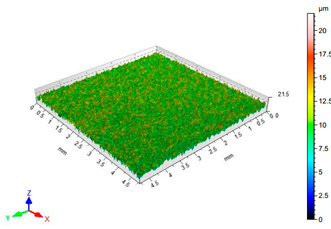	Sa = 1.31 μm Sz = 21.8 μm
9	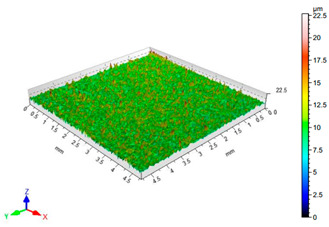	Sa = 1.02 μm Sz = 22.7 μm
10	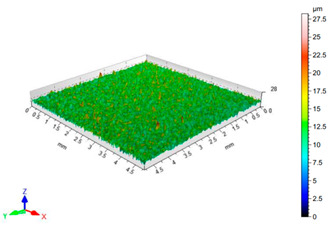	Sa = 1.51 μm Sz = 28.3 μm
11	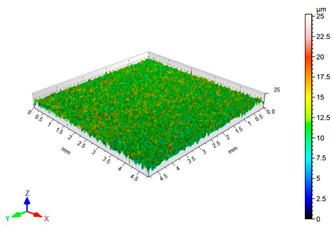	Sa = 1.58 μm Sz = 25.3 μm

**Table 9:** Droplet measuring the surface contact angles of EN AW 2024 T3 aluminium alloy sheet samples after sandblasting and values of the contact angles.

Number of Parameter sets Sandblasting	Droplet and Value of Contact Angle Measurement with Diiodomethane	Droplet and Value of Contact Angle Measurement with Water
reference surface	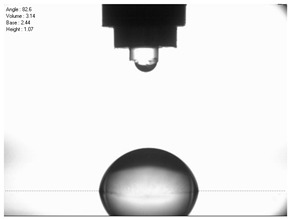 $\theta_{d}=79.11{}^{\circ}$	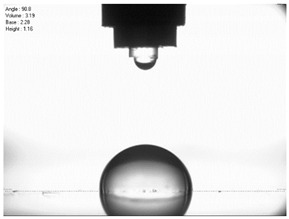 $\theta_{w}=89.74{}^{\circ}$
1	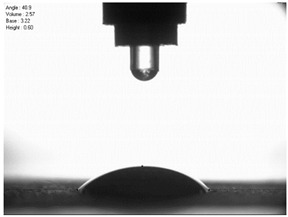 $\theta_{d}=38.52{}^{\circ}$	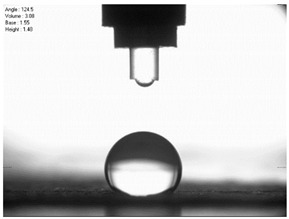 $\theta_{w}=117.75{}^{\circ}$
2	$\theta_{d}=47.72{}^{\circ}$	$\theta_{w}=101.06{}^{\circ}$
3	$\theta_{d}=44.10{}^{\circ}$	$\theta_{w}=118.85{}^{\circ}$
4	$\theta_{d}=43.20{}^{\circ}$	$\theta_{w}=107.87{}^{\circ}$
5	$\theta_{d}=40.47{}^{\circ}$	$\theta_{w}=123.95{}^{\circ}$
6	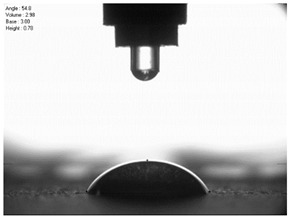 $\theta_{d}=57.62{}^{\circ}$	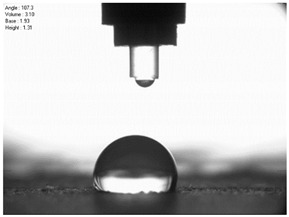 $\theta_{w}=113.28{}^{\circ}$
7	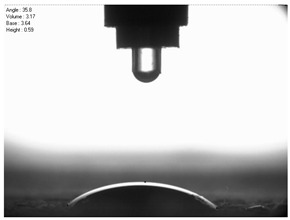 $\theta_{d}=37.05{}^{\circ}$	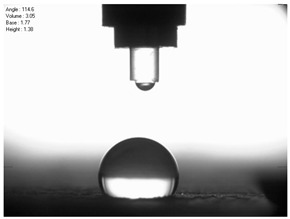 $\theta_{w}=113.88{}^{\circ}$
8	$\theta_{d}=43.96{}^{\circ}$	$\theta_{w}=107.70{}^{\circ}$
9	$\theta_{d}=43.85{}^{\circ}$	$\theta_{w}=101.90{}^{\circ}$
10	$\theta_{d}=45.20{}^{\circ}$	$\theta_{w}=113.12{}^{\circ}$
11	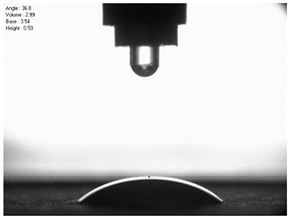 $\theta_{d}=45.70{}^{\circ}$	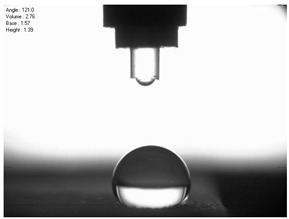 $\theta_{w}=115.56{}^{\circ}$

**Table 10:** Results of the W Shapiro–Wilk test for normality of the tensile shear strength distribution of EN AW 2024 T3 sheet adhesive joints as a function of sandblasting parameters.

Number of Parameter Sets Sandblasting	The Value of the *W* Shapiro–Wilk	Level *p* for the W Shapiro–Wilk
1	0.946	0.711
2	0.939	0.649
3	0.726	0.017
4	0.974	0.905
5	0.791	0.087
6	0.884	0.357
7	0.889	0.352
8	0.844	0.177
9	0.927	0.580
10	0.973	0.898
11	0.791	0.068

**Table 11:** Post hoc test results of homogeneous groups of mean tensile shear strength as a function of sandblasting parameters.

Number of Parameter Sets Sandblasting	Average Shear Strength R_t_ (MPa)	Homogeneous Groups				
		1	2	3	4	5
7	19.26	***				
1	17.68	***				
10	17.04	***	***			
3	13.87		***	***		
8	13.31			***		
5	13.13			***		
4	12.28			***	***	
11	12.22			***	***	
2	11.92			***	***	
6	8.84				***	***
9	7.60					***

Where: ***—indication of group assignment.

**Table 12:** Results of the correlation study.

Correlations Indicated Correlation Coefficients Are Significant with *p* < 0.05						
	Average	Standard Deviation	r (X,Y)	r^2^	t	*p*
Shear strength (MPa)	13.38	3.54				
Parameter Ra (μm)	1.19	0.27	0.57	0.33	2.10	0.07
Shear strength (MPa)	13.38	3.54				
Parameter Rz (μm)	9.40	1.96	0.39	0.15	1.27	0.24
Shear strength (MPa)	13.38	3.54				
Parameter Sa (μm)	1.27	0.29	0.53	0.29	1.90	0.09
Shear strength (MPa)	13.38	3.54				
Parameter Sz (μm)	23.21	5.07	0.67	0.44	2.68	0.03
Shear strength (MPa)	13.38	3.54				
SFE (mJ/m^2^)	38.65	3.48	0.58	0.34	2.14	0.06

Where r(X,Y)—Pearson correlation coefficient; r^2^—determination coefficient; t—value of the t statistic testing the significance of the correlation coefficient; *p*—the calculated significance level for the *t*-test.

## Data Availability

The raw/processed data required to reproduce these findings cannot be shared at this time due to technical or time limitations. Data can be made available on individual request.
